# *Toxoplasma gondii* in women of childbearing age and during pregnancy: seroprevalence study in Central and Southern Italy from 2013 to 2017

**DOI:** 10.1051/parasite/2019080

**Published:** 2020-01-14

**Authors:** Daniela Fanigliulo, Serena Marchi, Emanuele Montomoli, Claudia Maria Trombetta

**Affiliations:** 1 Department of Molecular and Developmental Medicine, University of Siena Via Aldo Moro 53100 Siena Italy; 2 VisMederi S.r.l. Strada del Petriccio e Belriguardo, 35 53100 Siena Italy

**Keywords:** *Toxoplasma gondii*, Seroprevalence, Women of childbearing age, Pregnancy, Italy

## Abstract

Toxoplasmosis is a worldwide health problem. Infection in pregnant women can result in severe fetal morbidity or in subclinical neonatal infection; most subclinical cases develop ocular and neurological sequelae. The purpose of this serological study was to assess the prevalence of *Toxoplasma gondii* in two populations of women of childbearing age in Siena (Tuscany, Central Italy) and Bari (Apulia, Southern Italy) between 2013 and 2017 and in a group of pregnant women in Bari in 2016–2017. Serum samples were tested for the presence of specific anti-*Toxoplasma gondii* IgG antibodies by a commercially available ELISA test. The percentage of seropositive subjects in Bari was significantly higher than in Siena (22.4% vs. 12.4%) and an age-related trend was observed. A low prevalence of *T. gondii* infection (13.8%) was observed among the pregnant women tested. In addition to showing a significant difference between Central and Southern Italy, this study provides updated data on *T. gondii* seroprevalence in women during childbearing age and pregnancy. The results confirm a trend toward a decrease, especially in younger people and pregnant women.

## Abstract (fr)

La toxoplasmose est un problème de santé mondial. L’infection d’une femme enceinte peut entraîner une morbidité fœtale grave ou une infection néonatale subclinique, et la plupart des cas subcliniques développeront des séquelles oculaires et neurologiques. Le but de cette étude sérologique était d’évaluer la prévalence de *Toxoplasma gondii* dans deux populations de femmes en âge de procréer à Sienne (Toscane, Italie centrale) et Bari (Pouilles, Italie du Sud) entre 2013 et 2017 et dans un groupe de femmes enceintes à Bari en 2016-2017. Des échantillons de sérum ont été testés pour la présence d’anticorps IgG anti-*Toxoplasma gondii* spécifiques par un test ELISA commercial. Le pourcentage de sujets séropositifs à Bari était significativement plus élevé qu’à Sienne (22,4 % vs 12,4 %) et une tendance liée à l’âge a été observée. Une faible prévalence de l’infection à *T. gondii* (13,8 %) a été observée chez les femmes enceintes testées. En plus de montrer une différence significative entre le centre et le sud de l’Italie, cette étude fournit des données actualisées sur la séroprévalence de *T. gondii* chez les femmes en âge de procréer et pendant la grossesse. Les résultats confirment une tendance à la baisse, notamment chez les jeunes et les femmes enceintes.

## Introduction

*Toxoplasma gondii* (*T. gondii*) is a protozoan parasite of worldwide distribution and one of the most common human zoonoses, infecting more than a third of the world’s population [17]. The parasite is horizontally transmitted to humans, mostly by ingestion or handling of water, food or soil contaminated with oocysts or raw or undercooked meat containing cysts. Infection is asymptomatic or associated with flu-like symptoms in more than 80% of immunocompetent individuals [25]. If primary infection occurs during pregnancy, *T. gondii* can cross the placenta and may be vertically transmitted to the fetus (congenital toxoplasmosis). Congenital toxoplasmosis may cause abortion, stillbirth or result in major ocular and neurological sequelae, ranging from slightly diminished vision to more severe disorders, such as retinochoroiditis, hydrocephalus, and intracerebral calcifications [15]. The risk of congenital infection and the severity of fetal damage depend on the gestational age when maternal infection occurs [25]. The overall risk of congenital infection from primary *T. gondii* infection during pregnancy ranges from 20% to 50% if untreated [10]. Congenital toxoplasmosis can be prevented by identifying non-immune women at the beginning of pregnancy, by providing information on how to avoid the infection, and by serological follow-up. Serological follow-up is based on repeated testing for specific IgG and IgM in order to distinguish, in the case of positivity, between acute and chronic infections [25]. In Italy, serological tests for toxoplasmosis are performed routinely during pregnancy, with the first evaluation carried out by the 13th week of pregnancy, followed by monthly testing of women who are seronegative for toxoplasmosis (total 5–7 tests) [16, 22].

*Toxoplasma gondii* seroprevalence rates increase with age; however, the rate of infection varies widely between countries and regions according to dietary habits, health standards and socioeconomic level. Improvements in hygiene conditions and farming systems, together with increased socioeconomic levels, have led to a declining seroprevalence in most industrialized countries [25]. As toxoplasmosis causes serious illness when it is vertically transmitted to the fetus during pregnancy, it has a significant impact on public health [12]. In Europe, nationwide epidemiological surveillance for congenital toxoplasmosis is currently implemented only in France and Germany. In Italy, the surveillance system for congenital toxoplasmosis, reporting data on live newborns from mothers with gestational toxoplasmosis, is active on a regional basis (e.g. Campania region) [27]. However, a nationwide epidemiological surveillance system for congenital toxoplasmosis is still lacking [1]. Consequently, no accurate estimates of the impact of toxoplasmosis on the population are available, and epidemiological information on the prevalence of *T. gondii* infection is incomplete, area-based, and mostly focused on pregnant women [3–5, 7, 8, 23, 24, 26, 28, 30]. Epidemiological data on *T. gondii* seroprevalence in women of childbearing age are even more scant [6, 11, 18, 20].

The aim of this study was to investigate the prevalence of *T. gondii* IgG antibodies in women of childbearing age in Tuscany (Central Italy) and Apulia (Southern Italy), and pregnant women in Apulia, in order to provide data on the current state of toxoplasmosis in Italy.

## Materials and methods

This study was performed on human serum samples collected from women of childbearing age (15–45 years old): 409 serum samples were collected in the province of Siena (Tuscany, Central Italy) in the years 2013, 2014, and 2016, and 398 in the province of Bari (Apulia, Southern Italy) from 2015 to 2016. In addition, 232 samples were collected from pregnant women in the province of Bari from 2016 to 2017. Serum samples were anonymously collected in compliance with Italian ethics law, and stored at the internal serum bank of the Laboratory of Molecular Epidemiology, Department of Molecular and Developmental Medicine, University of Siena.

A total of 1039 samples were tested for the presence of IgG antibodies against *T. gondii* “Enzywell Toxoplasma IgG” (DIESSE-Siena, Italy) commercial ELISA kits, in accordance with the manufacturer’s instructions. According to the manufacturer, the test offers 100% sensitivity and specificity; moreover, the presence of IgG antibodies against viruses such as Cytomegalovirus, Rubella, Epstein Barr, Herpes Simplex, and Mumps has no influence on the result of the test. On the basis of the test criteria, samples were classified as negative if the ratio between the optical density value of the sample and that of the cut-off was <0.7; positive if this ratio was >1.3. Samples with borderline results (ratio ≥0.7 or ≤1.3) were retested and, if still borderline when retested, excluded from the statistical analysis.

Samples were stratified by age groups (15–25, 26–35, and 36–45 years old) and prevalence rates were calculated along with their corresponding 95% confidence intervals (CI). Statistical analysis was performed by use of Yates’ corrected chi-square test and chi-square test for trends to compare prevalence rates among different groups and areas. Statistical significance was set at *p* < 0.05, two-tailed.

## Results

Of the 1039 serum samples tested, 19 had borderline results when retested, and were therefore excluded from the statistical analysis.

The overall anti-*T. gondii* IgG prevalence in samples collected from women of childbearing age in the province of Siena was 12.4% (9.5–16.0, CI), while in those collected in the province of Bari was 22.4% (18.6–26.8, CI) (*p* < 0.001) (Table 1). Significant differences were also found when comparing age groups. The prevalence in the 15–25-years-old age group was 4.2% (1.0–12.2, CI) in samples from Siena and 18.8% (12.1–27.8, CI) in samples from Bari (*p* < 0.01); in the 26–35-years-old age group, prevalence was 9.4% (5.4–15.5, CI) in Siena and 18.3% (13.2–24.9, CI) in Bari (*p* = 0.04); finally, the prevalence in the 36–45-years-old age group was 17.6% (12.8–25.8, CI) and 30.7% (23.3–39.2, CI) in Siena and Bari, respectively (*p* < 0.01). An increasing prevalence among age groups was found both in samples from Siena (*p* = 0.001) and Bari (*p* = 0.02).

**Table 1:** Prevalence of anti-*T. gondii* IgG in samples collected from women of childbearing age in Siena (2013, 2014, and 2016) and Bari (2015, 2016), divided by age groups (%, 95% CI; positive/total).

Age groups (years)	Siena	Bari
15–25	4.2 (1.0–12.2; 3/71)	18.8 (12.1–27.8; 18/96)
26–35	9.4 (5.4–15.5; 13/139)	18.3 (13.2–24.9; 31/169)
36-45	17.6 (12.8–25.8; 34/193)	30.7 (23.3–39.2; 39/127)
Total	12.4 (9.5–16.0; 50/403)	22.4 (18.6–26.8; 88/392)

The prevalence rate in samples collected from pregnant women was 13.8% (9.8–18.9, CI) (Table 2). No significant differences were found between age groups, while differences between pregnant women and women of childbearing age from Bari were found to be significant (*p* = 0.01), especially for the 36–45-years-old age group (*p* = 0.03).

**Table 2:** Prevalence of anti-*T. gondii* IgG in samples collected from pregnant women in Bari (2016–2017), divided by age groups (%, 95% CI; positive/total).

Age groups (years)	Bari
15–25	0.0 (0.0–48.9; 0/5)
26–35	13.0 (8.4–19.5; 19/146)
36–45	16.2 (9.4–26.4; 12/74)
Total	13.8 (9.8–18.9; 31/225)

## Discussion

In this study, the prevalence of anti-*T. gondii* antibodies in women of childbearing age from both the Tuscany and Apulia regions is consistent with the declining trend observed in recent decades in other regions in Italy [18, 20], as well as in other European countries [2, 9, 13] and the United States [14]. In Italy, *T. gondii* prevalence in women of childbearing age was 41.1% in the late 1980’s [6], and a trend toward a decrease has become evident since 2001 [20]. This decline in prevalence is correlated to declining incidence, due to lower exposure to the parasite by changes in nutritional habits and by improved hygiene practices in meat production [19].

The introduction of modern farming systems and the increase in consumption of frozen meat as the main factors for *T. gondii* incidence reduction are particularly evident in young people [13]. In fact, in this study, prevalence among age groups shows an age-related increase, with lower rates in younger women especially in samples collected in Siena.

As samples of this study were collected in two distinct geographic areas of Italy, differences in prevalence between Bari and Siena could be associated with sociodemographic and cultural factors [20], including different nutritional habits.

Prevalence in pregnant women is also consistent with this trend toward a decrease observed in Italy. Epidemiological studies conducted on *T. gondii* seroprevalence in pregnant women between the 1980’s and 1990’s revealed rates ranging from 40.4% in Naples to 48.7% in the area of Parma [3, 4, 30], whereas more recent studies have found rates of *T. gondii* prevalence in pregnant women between 21.5% and 27.5% [5, 7, 8, 26]. Pregnant women in this study showed a further decrease, with an overall prevalence of 13.8%. As for women of childbearing age, this decrease could be associated with improvements in quality of life and eating habits; however, prevalence in pregnant women was found to be significantly lower. This difference could be explained by the fact that pregnant women might be more aware of the specific hygiene and dietary recommendations to prevent primary *T. gondii* infection. In fact, the adoption of preventive measures due to a greater awareness of the risk associated with toxoplasmosis during gestation was already considered an explanation of lower *T. gondii* prevalence in women than in men [21], and it could be hypothesized that this kind of awareness is higher after counseling and education programs in antenatal clinics. In Italy, all pregnant women are strongly advised to undergo free-of-charge serological testing for toxoplasmosis. This testing is among the most important tools used to identify at-risk women and is considered a cost-effective means of prevention. For this reason, the diagnostic procedure is entirely covered by the public healthcare system [29].

This study is limited by a small sample size, in particular for samples from pregnant women, that were collected only in one geographical setting. However, it provides updated data on *T. gondii* seroprevalence in women of childbearing age and during pregnancy in two different regions of Italy, confirming a decreasing trend in the incidence of the infection.
